# Path Optimization Strategy for Unmanned Aerial Vehicles Based on Improved Black Winged Kite Optimization Algorithm

**DOI:** 10.3390/biomimetics10050310

**Published:** 2025-05-11

**Authors:** Shuxin Wang, Bingruo Xu, Yejun Zheng, Yinggao Yue, Mengji Xiong

**Affiliations:** 1 School of Intelligent Manufacturing, Shanghai Zhongqiao Vocational and Technical University, Shanghai 201514, China; 2 School of Intelligent Manufacturing and Electronic Engineering, Wenzhou University of Technology, Wenzhou 325035, China; 3 Engineering Technology Department, Shanghai Caoyang Vocational School, Wenzhou 200333, China

**Keywords:** Black-winged Kite Optimization Algorithm, tent chaos mapping, Gaussian mutation, UAV path planning

## Abstract

The Black-winged Kite Optimization Algorithm (BKA) is likely to experience a sluggish convergence rate when confronted with the optimization of complex multimodal functions. The fundamental algorithm has a tendency to get stuck in local optima, thus rendering it arduous to identify the global optimal solution. When dealing with large-scale data or high-dimensional optimization challenges, the BKA algorithm entails significant computational expenses, which might lead to excessive memory usage or prolonged running durations. In order to enhance the BKA and tackle these problems, a revised Black-winged Kite Optimization Algorithm (TGBKA) that incorporates the Tent chaos mapping and Gaussian mutation strategies is put forward. The algorithm is simulated and analyzed alongside other swarm intelligence algorithms by utilizing the CEC2017 test function set. The optimization outcomes of the test functions and the function convergence curves indicate that the TGBKA demonstrates superior optimization precision, a quicker convergence speed, as well as robust anti-interference and environmental adaptability. It is also contrasted with numerous similar algorithms via simulation experiments in various scene models for Unmanned Aerial Vehicle (UAV) path planning. In comparison to other algorithms, the TGBKA produces a shorter flight route, a higher convergence speed, and stronger adaptability to complex environments. It is capable of efficiently addressing UAV path planning issues and improving the UAV’s path planning abilities.

## 1. Introduction

In human production and daily life, there exists a vast number of optimization problems. Achieving successful optimization can cut down on costs and increase benefits [1,2]. At present, traditional optimization algorithms have been extensively utilized in domains including engineering design, production planning and scheduling, as well as economic and financial analysis, and have achieved satisfactory results [3,4]. Nevertheless, with the rapid advancement of science and technology, complex optimization problems, such as high-dimensional, non-linear, and multi-objective ones, are becoming increasingly common in engineering, economics, artificial intelligence, and other areas [5]. When dealing with complex environments and large-scale problems, traditional optimization algorithms encounter restrictions in terms of efficiency and accuracy [6,7]. Hence, it is of utmost importance to discover appropriate optimization methods. Swarm intelligence algorithms have offered significant aid in fields such as engineering applications [8,9], intelligent healthcare [10,11], and financial investment [12,13]. Metaheuristic Algorithms are a category of advanced optimization algorithms grounded in heuristic strategies, which are employed to solve complex, high-dimensional, multimodal, or dynamic optimization problems [14]. Unlike traditional mathematical programming approaches, metaheuristic algorithms do not depend on the gradient information of the objective function. Instead, they create global search mechanisms by imitating natural phenomena, biological behaviors, or physical laws, enabling them to escape local optima and approach the global optimal solution. Their key characteristics include generality, which allows them to be applied to various optimization scenarios without being tailored to a specific problem, global search capabilities that avoid premature convergence by randomly exploring the solution space; and adaptability to flexibly adjust search strategies [15].

The above-mentioned research background suggests that the enhancement and optimization of swarm intelligence algorithms and metaheuristic algorithms are of great significance for the following reasons [16]. First of all, optimizing the basic algorithms can surmount the inherent flaws of swarm intelligence algorithms and metaheuristic algorithms, enhance global search abilities, convergence rate, and computational efficiency, and strengthen the robustness and generality of the algorithms, thus improving their adaptability to different problems and situations [17]. Secondly, optimizing the basic algorithms can help meet the requirements of complex real-world problems. Current practical scenarios are more diversified, and during the actual planning process, it is often necessary to optimize multiple objectives concurrently, such as, but not limited to, costs, time, and energy consumption [18]. Optimization efforts should be made to pursue maximum benefits. Thirdly, optimizing the basic algorithms can drive the innovation of algorithm theory and applications. In the process of algorithm improvement, it is frequently necessary to draw on disciplines like physics and sociology, which promotes interdisciplinary research [19]. In cutting-edge fields such as artificial intelligence and the Internet of Things, efficient optimization technologies are crucial, and the improvement outcomes of algorithms directly determine the technical implementation effect and economic value [20]. To address these issues, the Tent-Gaussian-Based Black-winged Kite Optimization Algorithm (TGBKA) is proposed in this study. The key idea behind TGBKA lies in the integration of two effective strategies. First, the Tent chaos mapping is introduced during the population initialization stage of the black-winged kite algorithm. The Tent chaos mapping can generate a more evenly distributed initial population, which enhances the diversity of the population and improves the algorithm’s exploration ability at the beginning of the search process [21]. This helps the algorithm to cover a larger portion of the solution space and avoid getting trapped in local optima from the start. Second, Gaussian mutation is incorporated into the migration stage of the black-winged kites. Gaussian mutation allows the algorithm to introduce random changes to the solution in a more intelligent way. It helps the algorithm escape from local optima by exploring regions of the solution space that are far from the current position. At the same time, it also maintains a certain degree of local search ability, ensuring that the algorithm can refine the solution once it approaches a promising region. By combining these two strategies, TGBKA aims to overcome the limitations of the basic BKA and other existing algorithms, improving its performance in terms of optimization speed, accuracy, and the ability to avoid local optima, especially in complex optimization problems.

## 2. Related Work

Swarm intelligence algorithms have a rich research history and a wide range of applications. The Particle Swarm Optimization (PSO) algorithm, one of the most well-known swarm intelligence algorithms, was inspired by the social behavior of birds flocking or fish schooling. In PSO, each particle represents a potential solution in the search space, and particles update their positions based on their own best-known position and the best-known position in the entire swarm [22]. PSO has been successfully applied to various fields, such as function optimization, neural network training, and power system optimization. However, PSO also has some drawbacks, such as premature convergence, especially in complex multimodal problems. The Ant Colony Optimization (ACO) algorithm is another prominent swarm intelligence algorithm, inspired by the foraging behavior of ants. ACO uses artificial ants to construct solutions by depositing pheromones on the paths they traverse. Over time, paths with better solutions tend to accumulate more pheromones, guiding other ants to follow these paths. ACO has been widely used in combinatorial optimization problems, such as the traveling salesman problem, vehicle routing problem, and job-shop scheduling problem. But it often requires a large number of iterations to converge, and its performance may degrade in large-scale problems [23].

Metaheuristic algorithms cover a broad spectrum of optimization techniques. The Genetic Algorithm (GA), a classic metaheuristic algorithm, is based on the principles of natural selection and genetics. GA uses operators such as selection, crossover, and mutation to evolve a population of candidate solutions over generations. It has been applied to diverse areas, including engineering design, data mining, and machine learning. However, GA may have difficulty in fine-tuning the solution in the later stages of the search process and may also suffer from premature convergence. The Simulated Annealing (SA) algorithm, inspired by the annealing process in physics, allows the algorithm to accept worse solutions with a certain probability at the beginning of the search, which helps it escape from local optima. As the search progresses, the probability of accepting worse solutions gradually decreases. SA has been used in problems such as network design, VLSI layout, and image processing. But its performance highly depends on the cooling schedule, and improper setting of the cooling schedule can lead to slow convergence or failure to find the optimal solution [24].

The Black-winged Kite Optimization Algorithm (BKA) is a relatively new addition to the family of swarm intelligence algorithms. It simulates the behaviors of black-winged kites attacking prey and migrating to perform optimization. The basic BKA has shown some potential in solving optimization problems, but as mentioned earlier, it has limitations in population diversity and local-optimum avoidance. Some variants of BKA have been proposed in the literature to address these issues. For example, researchers have tried to modify the attack and migration strategies of BKA or combine it with other algorithms. However, these existing variants have not fully overcome the inherent problems of the basic BKA, and there is still room for improvement.

In summary, while existing swarm intelligence and metaheuristic algorithms have made significant contributions to the field of optimization, they still face various challenges in handling complex optimization problems. The proposed TGBKA aims to build on the existing research and provide a more effective solution to these problems.

## 3. UAV Path Optimization Mathematical Model

### 3.1. Terrain Area Modeling

UAVs need to execute flight missions in a complex three-dimensional space. When simulating UAV flight missions, a three-dimensional environment model is required [25]. $P_{ij}=\left(x_{ij},y_{ij},z_{ij}\right)$, represents the track position of the *j*-th path point in the *i*-th flight path of the UAV, and its flight space can be expressed as [26]:

(1) $$\left\{P_{ij}=\left(x_{ij},y_{ij},z_{ij}\right)\left|\begin{array}{l} x_{\min}\leq x_{ij}\leq x_{\max}\\ y_{\min}\leq y_{ij}\leq y_{\max}\\ z_{\min}\leq z_{ij}\leq z_{\max} \end{array}\right\}\right.$$

where $x_{\min}$, $x_{\max}$, $y_{\min}$, $y_{\max}$, $z_{\min}$, and $z_{\max}$ represent the boundaries of the flight space, respectively, which restrict the coordinate range of the path nodes to ensure that the path does not exceed the map boundaries and altitude limits [27,28].

### 3.2. Threat Area Modeling

In this experiment, the threat area is defined as a cylinder to simulate no-fly zones or dangerous areas.

(2) $$\sqrt{{\left(x-x_{i}\right)}^{2}+{\left(y-y_{i}\right)}^{2}}\;\leq R_{i},\;z\in \left[z_{i}-\Delta ,z_{i}+\Delta \right]$$

where $\left(x_{i},y_{i},z_{i}\right)$ is the center of the cylinder, *r* is the radius, and *h* is the height tolerance.

### 3.3. Loss Function

After modeling the flight space, it is necessary to comprehensively consider the constraints, such as the UAV path length cost, altitude cost, and path smoothness cost. Based on the above conditions, the flight cost function is expressed as [29]:

(3) $$C=\omega_{1}C_{path}+\omega_{2}C_{height}+\omega_{3}C_{turn}$$

where *C* is the total flight path loss function, $C_{path}$ represents the UAV path cost, $C_{height}$ represents the UAV flight altitude cost, $C_{turn}$ represents the UAV turning cost, and $\omega_{i}$ is the weights of the cost function [30]. *ω*_1_ = 0.5, *ω*_2_ = 0.3, and *ω*_3_ = 0.2. In this experiment, the cost priority is set as: altitude constraint > path length > smoothness.

## 4. Black-Winged Kite Optimization Algorithm

In the fundamental Black-winged Kite Optimization Algorithm (BKA), the characteristic behaviors of black-winged kites, namely attacking prey and migrating, are meticulously simulated [31]. The action of attacking prey can be regarded as an iterative process of exploring and identifying the optimal solution within a predefined search range. In the context of the BKA, the prey serves as an abstract representation of the global optimal solution that the algorithm aims to converge to [32,33]. This aspect mimics how black-winged kites zero in on their targets in the natural world, translating this predatory behavior into a search mechanism for the best possible solution in the computational domain.

On the other hand, the migration behavior of black-winged kites is emulated as a strategic operation equivalent to conducting a search over a much larger area. This emulation is of great significance because it endows the algorithm with the ability to explore different regions of the solution space. By doing so, it effectively prevents the algorithm from prematurely converging and getting trapped in local optima, which is a common pitfall in many optimization algorithms. During the process of seeking the optimal solution, this migration-like exploration ensures a more comprehensive search, increasing the likelihood of discovering the true global optimum [34].

**Figure 1:**
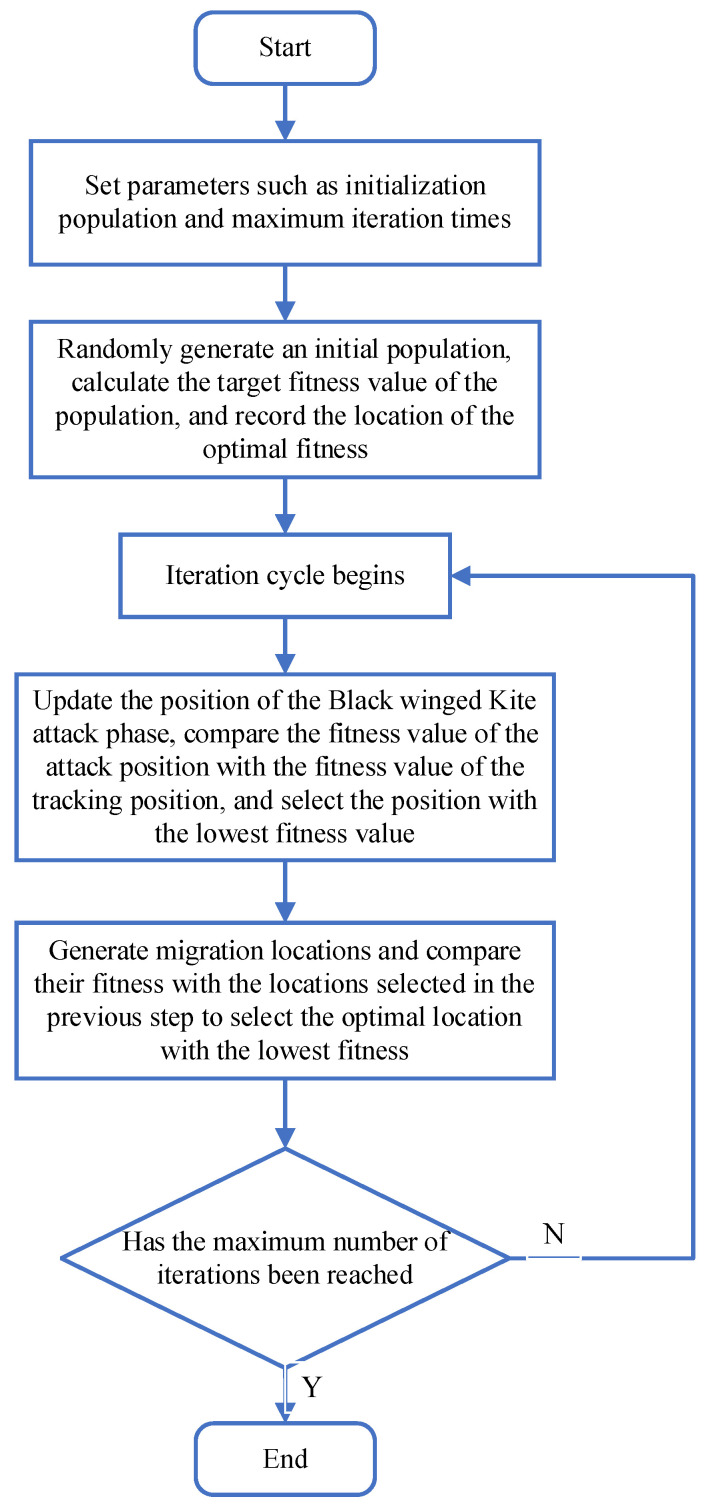
The flowchart of the basic black-winged kite optimization algorithm.

The BKA unfolds in a sequential manner and can be systematically divided into three distinct and crucial stages: the population initialization stage, the attack stage, and the migration stage. The population initialization stage sets the foundation by randomly generating an initial set of candidate solutions, which represent the initial positions of the black-winged kites in the search space. This initial population then progresses to the attack stage, where the kites, metaphorically speaking, attempt to locate and approach the prey (optimal solution) within their immediate vicinity. Finally, in the migration stage, the kites move to new locations, enabling the algorithm to explore uncharted areas of the solution space. The basic flowchart of the fundamental Black-winged Kite Optimization Algorithm, as depicted in Figure 1, provides a visual representation of these stages and the overall flow of the algorithm, helping to better understand its operational mechanism and sequential execution.

### 4.1. Population Initialization

The initialization stage of the BKA usually uses random initialization. The first step of initialization is to create a set of random solutions. The matrix in Equation (4) can be used to represent the position of each black-winged kite [35]:

(4) $$BK=\left[\begin{array}{ccccc} BK_{1,1} & BK_{1,2} & \ldots & \ldots & BK_{1,din}\\ BK_{2,1} & BK_{2,2} & \ldots & \ldots & BK_{2,din}\\ \vdots & \vdots & \vdots & \vdots & \vdots \\ \vdots & \vdots & \vdots & \vdots & \vdots \\ BK_{pop,1} & BK_{pop,2} & \ldots & \ldots & BK_{pop,din} \end{array}\right]$$

where pop is the number of potential solutions, dim is the dimension of the given problem, $BK_{ij}$ is the *j*-th dimension of the *i*-th black-winged kite. The positions of each black-winged kite are uniformly distributed as follows [36]:

(5) $$X_{i}=BKA_{lb}+rand(BKA_{\mathrm{ub}}-BKA_{lb})$$

In Equation (5), the parameter *N* is the size of the black-winged kite population. $X_{i}$ is the position of the *i*-th black-winged kite, that is, the current single solution. the parameter rand is a random number between 0 and 1. $BKA_{lb}$ and $BKA_{\mathrm{ub}}$ represent the lower and upper bounds of the position of the *i*-th black-winged kite in the *j*-th dimension respectively.

During the initialization process, the BKA algorithm selects the individual with the best fitness as the leader in the initial population. The mathematical expressions for initializing the $X_{L}$ are shown in Equations (6) and (7), taking the minimum value as an example:

(6) $$f_{best}=\min\left(f\left(X_{i}\right)\right)$$

(7) $$X_{L}=X\left(find\left(f_{best}==f\left(X_{i}\right)\right)\right)$$

### 4.2. Attack Stage

The black-winged kites, which prey on small grassland mammals and insects, exhibit remarkable flight maneuvers. During their flight, they skillfully adapt the angles of their wings and tails in response to the changing wind speed. This dynamic adjustment allows them to maintain a stable and silent hover, enabling them to carefully observe their surroundings for potential prey. Once they have identified a target, they execute a rapid and precise dive, swooping down to launch an attack. This hunting strategy is not only a display of their natural predatory prowess but also serves as an inspiration for algorithmic design. In the context of optimization algorithms, these different attack behaviors can be analogized to various search strategies for global exploration. By mimicking the black-winged kites’ ability to survey a wide area during hovering and then zero in on specific targets during the dive, algorithms can be designed to efficiently explore the solution space, balance the search between broad exploration and focused exploitation, and ultimately enhance their effectiveness in finding optimal solutions [37].

(8) $$X_{t+1}^{i,j}={\{}_{X_{t}^{i,j}+n\left(2r-1\right)\times X_{t}^{i,j}\;\;\;\;\;\;\;\;\;\;\;\;\;\;\;\;\mathrm{else}}^{X_{t}^{i,j}+n\left(1+\sin\left(r\right)\times X_{t}^{i,j}\right)\;\;\;\;\;\;\;\;\;\;p<r}$$

(9) $$n=0.05\times {e^{-2\times \left(\frac{t}{T}\right)}}^{2}$$

where Equation (8) simulates the scenarios of black-winged kites hovering and spreading their wings in the air to maintain balance and quickly rushing towards prey. Equation (9) simulates the attack state of black-winged kites when hovering in the air and their hovering state. In Equation (8), $X_{t+1}^{i,j}$ and $X_{t}^{i,j}$ represent the positions of the *i*-th black-winged kite in the *j*-th dimension at the *t*-th and *t* + 1-th iterations, respectively. The parameter *r* is a random number between 0 and 1, and the parameter p is a constant, p = 0.9. In Equation (9), the parameter *T* is the maximum number of iterations, that is, the total number of iterations, and the parameter *t* is the current iteration number [38].

### 4.3. Migration Stage

Bird migration is a remarkable phenomenon driven by the need to adapt to seasonal fluctuations. As winter approaches, numerous bird species undertake long-distance journeys from the north to the south. This mass movement is primarily influenced by environmental factors such as harsh climates and scarce food resources in their northern habitats. By migrating to more favorable southern regions, birds can secure better living conditions and access abundant food supplies, thereby increasing their chances of survival and reproduction. During migration, a hierarchical structure often emerges, with leaders playing a pivotal role. These leaders possess highly developed navigation abilities, which are essential for guiding the flock safely to their destination. Their skills in sensing celestial cues, Earth’s magnetic fields, and other environmental markers ensure that the group stays on course and avoids getting lost.

In the Black-winged Kite Optimization Algorithm (BKA), the concept of bird migration serves as a rich source of inspiration. The algorithm formulates a hypothesis that mirrors the decision-making process in avian migration. Specifically, it assesses the fitness values of different populations within the algorithmic framework. When the fitness value of the current population falls short of that of a randomly generated population, it implies that the current leader lacks the necessary qualities to effectively lead the group forward. In such a situation, the leader relinquishes its leadership position and integrates back into the migrating population. On the contrary, when the fitness value of the current population surpasses that of the random population, the leader is deemed capable and proceeds to guide the population towards the desired destination. This dynamic leadership selection mechanism in the BKA algorithm ensures that only the most competent leaders are entrusted with the task of leading the “population” of solutions, thereby enhancing the algorithm’s ability to navigate through the solution space and achieve successful “migration” towards optimal or near-optimal solutions [39,40].

(10) $$X_{t+1}^{i,j}={\{}_{X_{t}^{i,j}+C\left(0,1\right)\times \left(L_{t}^{j}-m\times X_{t}^{i,j}\right)\;\;\;\;\;\;\;\mathrm{else}}^{X_{t}^{i,j}+C\left(0,1\right)\times \left(X_{t}^{i,j}-L_{t}^{j}\right)\;\;\;\;\;\;\;\;\;\;F_{i}<F_{ri}}$$

(11) $$m=2\sin\left(r+\pi /2\right)$$

where $L_{t}^{j}$ represents the leading scorer of black-winged kites in the *j*-th dimension at the *t*-th iteration so far, that is, the current optimal solution. $F_{i}$ represents the fitness value of the current black-winged kite individual. $F_{ri}$ represents the fitness value of a randomly selected black-winged kite’s position in the *j*-th dimension at the *t*-th iteration. $C\left(0,1\right)$ represents the Cauchy mutation, and its definition is as follows:

The one-dimensional Cauchy distribution is a continuous probability distribution with two parameters. The probability density function of the one-dimensional Cauchy distribution is:

(12) $$f\left(x,\delta ,\mu \right)=\frac{1}{\pi }\frac{\delta }{\delta^{2}+{\left(x-\mu \right)}^{2}}\;\;\;\;\;\;-\infty <x<\infty$$

when μ = 0 and δ = 1, the probability density function becomes the standard form, and the formula is as follows:

(13) $$f\left(x,\delta ,\mu \right)=\frac{1}{\pi }\frac{1}{x^{2}+1}\;\;\;\;\;\;-\infty <x<\infty$$

## 5. Improved Black-Winged Kite Optimization Algorithm

Considering the issues of the Black-winged Kite Optimization Algorithm (BKA), such as its slow convergence rate and tendency to be trapped in local optima, this paper employs the Tent chaos mapping during the population initialization stage. This approach aims to make the initial solutions spread as evenly as possible across the solution space. Additionally, Gaussian mutation is introduced in the migration stage to boost the algorithm’s convergence speed. Regarding the naming, the first letters of “Tent Map” and “Gaussian Mutation” (GM) are combined. Consequently, the enhanced Black-winged Kite Optimization Algorithm proposed in this study is named TGBKA.

### 5.1. Tent Chaos Mapping

Numerous optimization algorithms have demonstrated that chaos mapping is highly suitable for initializing the population of these algorithms. Chaos mappings encompass various types, including Logistic mapping, Cubic mapping, Sine mapping, and Tent mapping [41], among others. Nevertheless, the population generated by Logistic mapping is unevenly distributed, often clustering in the middle or at the two ends. As a result, the initial population covers only a limited portion of the solution space [42]. Cubic mapping also exhibits a bias towards the two ends in its distribution, with inadequate coverage, which may reduce the initialization efficiency when dealing with high-dimensional optimization problems. For Sine mapping, the chaos parameter must be precisely controlled within a specific range to maintain the chaotic state. In practical applications, however, there are certain constraints on the flexibility of parameter adjustment [43].

In contrast, the Tent mapping generates a chaotic sequence that is more uniformly distributed within the interval (0,1). Notably, when the parameter is set to 0.5, the linear piece-wise structure of the Tent mapping guarantees ergodicity. This characteristic allows it to effectively cover the solution space and enhance the diversity of the initial population. Furthermore, the Tent mapping is less sensitive to parameter changes. Even if the parameters vary slightly within the range of (0, 1), it can still retain good chaotic properties, endowing it with broader applicability [44]. Therefore, for the enhancement of the black-winged kite algorithm during the population initialization stage, this paper selects the Tent chaos mapping. By using the Tent chaos mapping, the basic Black-winged Kite Optimization Algorithm can generate high-quality initial solutions with excellent diversity in the search space. A high-quality initial population significantly contributes to improving the subsequent convergence speed and optimization performance of the algorithm.

(14) $$x_{i+1}={\{}_{(1-x_{i})\times \mu \;\;\;\;,x_{i}\geq 0.5}^{\;\;\;\;x_{i}\times \mu \;\;\;\;\;\;\;,x_{i}<0.5}$$

where $x_{i+1}$ represents the position after mapping. $x_{i}$ represents the original position of the target. The parameter *i* represents the population size. μ∈[0,2], and the larger the value of μ, the better the chaotic performance.

### 5.2. Gaussian Mutation

Gaussian Mutation (GM) is an optimization approach where random numbers conforming to a normal distribution are applied to the original position vector to create new positions [45]. The majority of mutation operators are centered around the original position, effectively performing a local search within a limited area [46]. This form of mutation not only boosts the optimization precision of the algorithm but also enables it to break free from local optimal solutions [47]. Additionally, some operators deviate significantly from the current position, increasing population diversity and enabling more effective exploration of potential areas. This, in turn, improves the search efficiency and speeds up the convergence of the optimization algorithm [48]. The Gaussian probability density formula is presented below:

(15) $$f(x)=\frac{1}{\sigma \sqrt{2\pi }}e^{-\frac{1}{2}{\left(\frac{x-\mu }{\sigma }\right)}^{2}}$$

In Equation (15), the parameter μ represents the mean or expected value of the distribution, determining the central position of the distribution, and the curve is symmetric about x=μ. The parameter σ represents the standard deviation, determining the width or degree of dispersion of the distribution. The larger σ is, the wider and flatter the curve is. The smaller σ is, the narrower and taller the curve is. The first part serves as a normalization factor to ensure that the integral of the entire probability density function is 1. The second part is used to control the shape of the distribution. As *x* gradually deviates from μ, the exponential part decays rapidly and gradually approaches 0, forming a “bell-shaped curve”.

After Gaussian mutation improvement, there are usually two update forms: additive mutation in Equation (16), which is to superimpose perturbations on the original solution, and replacement mutation in Equation (17), which is to directly generate new values. This paper adopts the replacement mutation in Equation (17).

(16) mx=x+σ×N(0,1)

(17) $$mx∼N(\mu ,\sigma^{2})$$

Combined with the mathematical model of the above-mentioned Black-winged Kite Optimization Algorithm, the execution steps of TGBKA can be summarized into the following five steps:

Step 1: Enter the population size, the iteration count, the lower limit, the upper limit, and the dimension of the algorithm. By means of Equation (15), incorporate the Tent chaos mapping to initialize the black-winged kite population.

Step 2: Compute the fitness of every individual based on the positions within the black-winged kite population. Sort the population in accordance with the fitness values, and identify the current best individual as the leader.

Step 3: Iterate through all the individuals. Carry out the attack behavior as per Equations (8) and (9). Assess the fitness of the new position, and decide whether to update the individual’s position based on the fitness result.

Step 4: Iterate through all the individuals. Execute the migration behavior according to Equations (10), (11), (16), and (17). Evaluate the fitness of the new position, and determine whether to update the individual’s position based on the fitness.

Step 5: Check whether the algorithm has reached the maximum number of iterations. If it has, update and output the global optimal solution; if not, go back to Step 3.

The algorithmic pseudocode of the enhanced Black-winged Kite Optimization Algorithm TGBKA is presented in Algorithm 1.
**Algorithm 1.** Algorithm Pseudocode of TGBKA.Input: Population size *pop* Number of iterations *T* Lower bound *lb* Upper bound *ub* Dimension dim Objective function *fobj*1. Initialize the black-winged kite population.2. *Set p = 0.9 and calculate the initial fitness*.3. For *t* = 1 to *T*:4. Calculate the fitness of each individual, sort the population according to the fitness, and obtain the current optimal solution.5.  For each individual *i* = 1 to *pop*:6.    Execute the attack behavior.7.    Execute the migration behavior.8.  End for9.  Update the global optimal.10. End forOutput: Best fitness *Best_Fitness_BKA*, best position *Best_Pos_BKA*.

**Figure 2:**
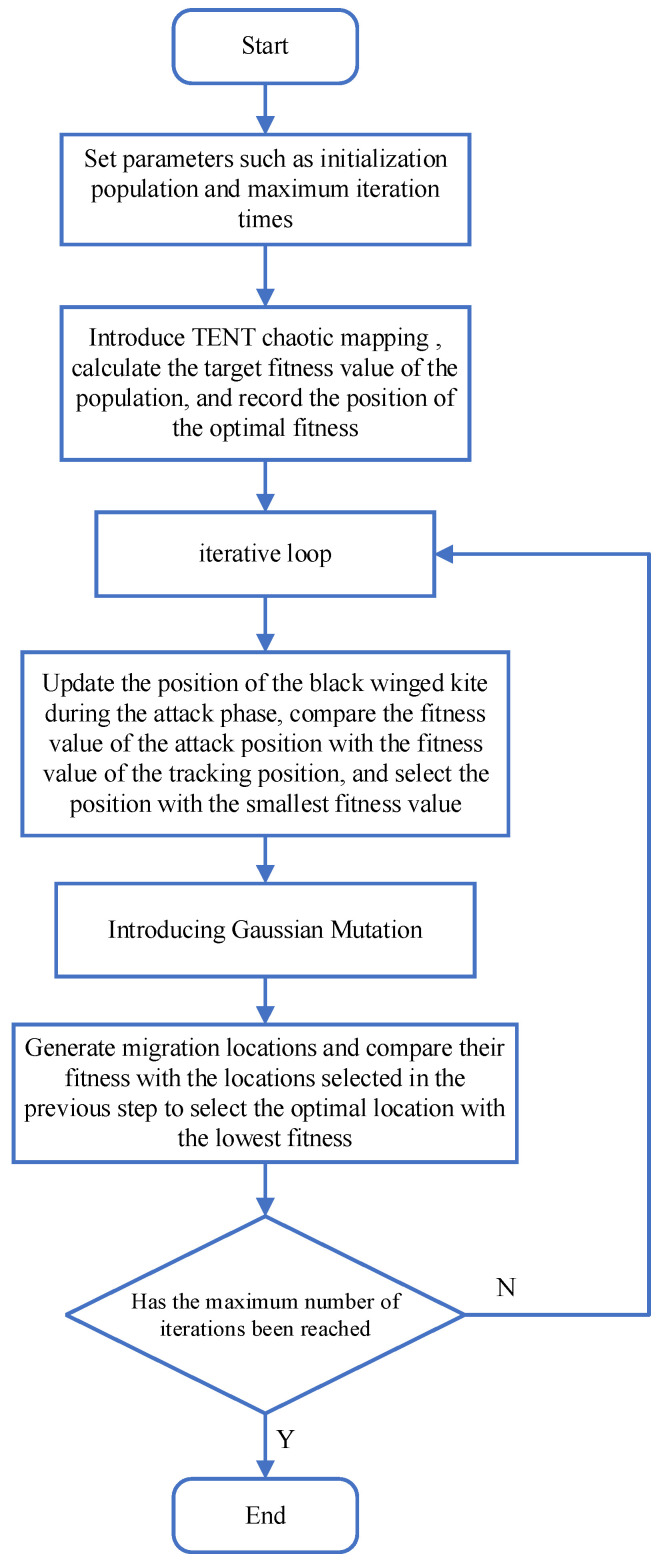
Flowchart of the Improved TGBKA Algorithm.

The flowchart of the improved Black-winged Kite Optimization Algorithm TGBKA is shown in Figure 2.

## 6. Experimental Results and Analysis of the Algorithm Test

### 6.1. Experimental Simulation Environment

In order to enhance the performance of the Black-winged Kite Optimization Algorithm (BKA), this paper puts forward two improvement strategies. Firstly, the Tent chaos mapping is employed to initialize the population in the basic Black-winged Kite Optimization Algorithm. Secondly, Gaussian mutation is introduced during the migration stage of the black-winged kites. The effectiveness of these improvement strategies requires verification through experiments. Thus, in this section, the TGBKA algorithm will be contrasted with the five algorithms previously mentioned, namely the BKA, SCGJO [49], COA [50], SABO [51], and HHO [52]. This comparison will be carried out via numerical experiments on 29 functions from the CEC2017 benchmark test set. The CEC2017 benchmark has a high level of popularity and wide recognition in related fields, and its use can facilitate comparison and evaluation with other research results. To guarantee the fairness of the experiment, the common parameters of all algorithms are set uniformly: the dimension is set as *dim* = 30, the population size is *N* = 30, and the maximum number of iterations is *Max_iter* = 500. The optimal value Min, the average error value Mean, and the standard error value Std obtained from 30 independent experiments are taken as the experimental outcomes.

### 6.2. Performance Indicators

In order to comprehensively analyze the performance indicators of various optimization algorithms during the experiment and select the optimal algorithm, three indicators, namely the optimal value *Min*, average error value *Mean*, and standard error value *Std*, are selected for algorithm performance evaluation. Their definitions are as follows. Here, *m* is the number of runs.

1. Optimal value *Min*.

*Min* is the best result obtained from the *i*-th run of the algorithm.

2. Average error value *Mean*.

(18) $$Mean=\frac{1}{m}\sum_{i=1}^{m}Error_{i}\;\;\;\;,Error_{i}=\left(best_{i}-M\mathrm{in}\right)$$

3. Standard error value *Std*.

(19) $$Std=\sqrt{\frac{1}{m-1}\sum_{i=1}^{m}{\left(Error_{i}-Mean\right)}^{2}}$$

### 6.3. Comparison of Algorithms in the CEC2017 Test Set

In the CEC2017 test set, the minimum value Min of TGBKA ranked first in 24 benchmark test functions, demonstrating excellent minimization optimization performance. This shows that the introduction of Gaussian mutation in the basic Black-winged Kite Optimization Algorithm significantly improves the optimization accuracy and search speed of BKA. The average error value Mean and standard error value Std of TGBKA are smaller than those of the other 5 algorithms in 21 functions and 16 functions, respectively, reflecting the excellent stability performance of TGBKA. The comparison of the optimization results of the test functions is shown in Table 1.

Specifically, for unimodal functions F1–F3, the Mean value of TGBKA when solving F3 has an order-of-magnitude improvement, and it ranks second only to HHO when solving F1. For multimodal functions F4–F10, TGBKA is not the best only in F7, but its order of magnitude is at the same level. For hybrid functions F11–F20, TGBKA fails to obtain the optimal value only in one case, indicating that the introduction of the Gaussian mutation strategy can effectively improve the optimization accuracy of the algorithm. For composite functions F21–F30, TGBKA obtains the best optimization results in most functions.

In conclusion, the combination of using the TENT chaos mapping to initialize the population and Gaussian mutation to optimize the migration stage in the basic black-winged kite optimization algorithm is effective, which greatly improves the performance of the basic BKA algorithm.

**Figure 3:**
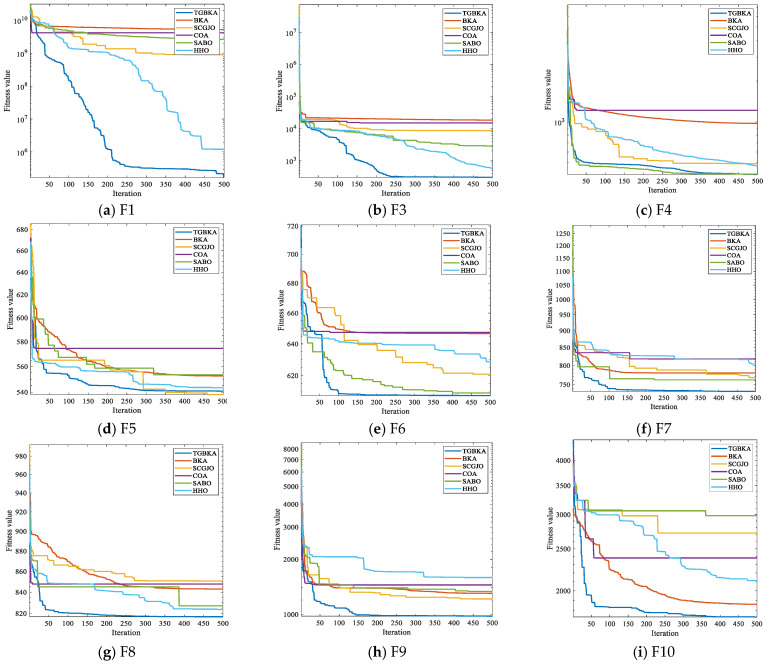

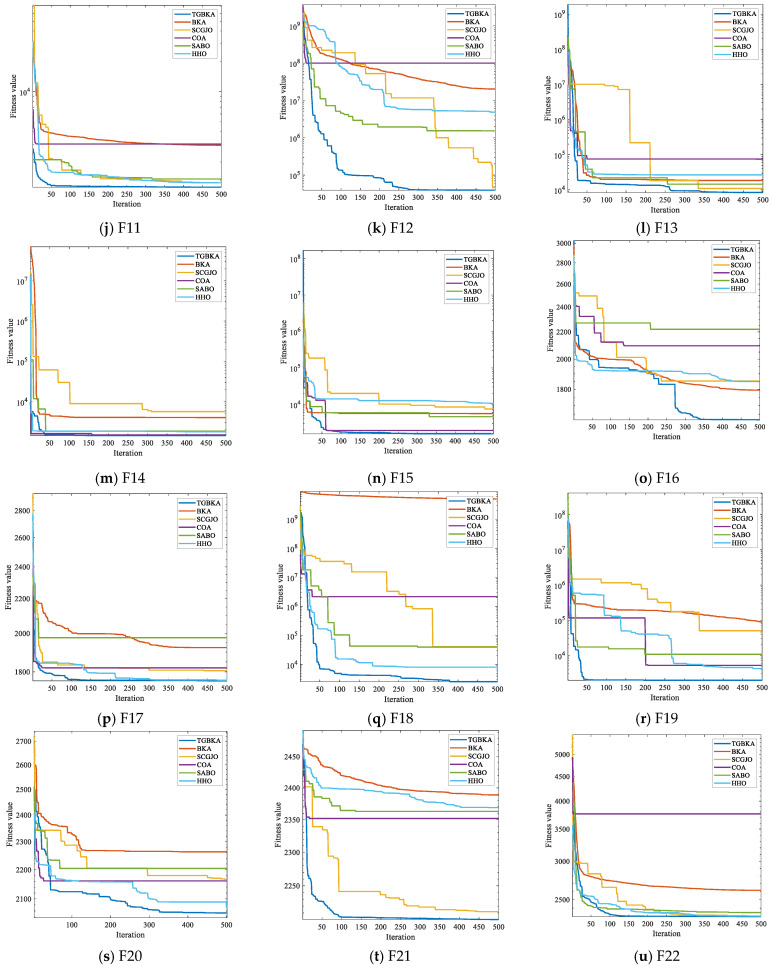

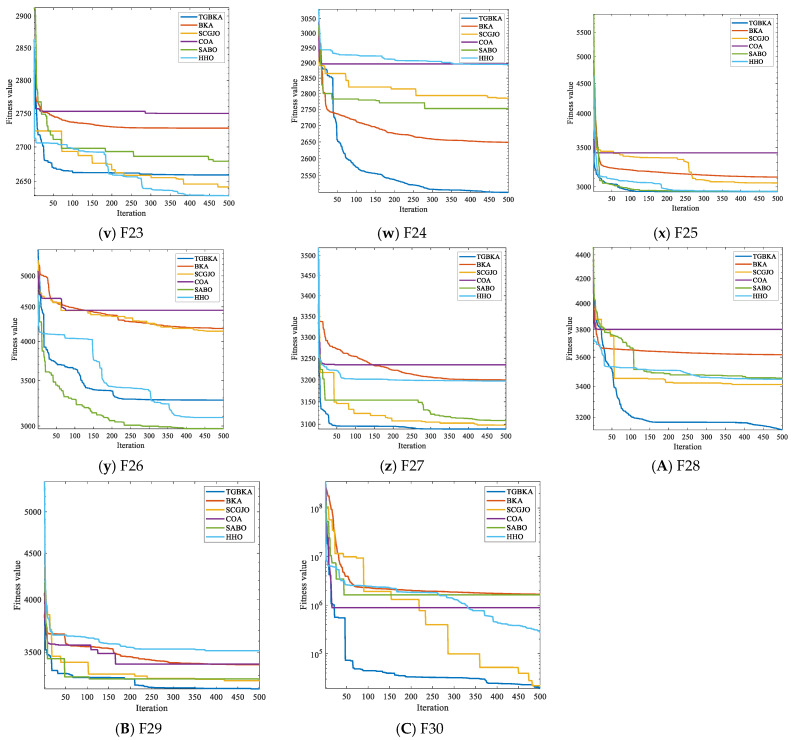
Convergence Curves of Test Function Algorithms.

To more clearly observe the contribution of the optimization scheme proposed in this paper to the optimization performance of BKA and to more intuitively compare the differences in the optimization accuracy and convergence performance of the algorithms on the test functions, Figure 3 shows the convergence curves of six algorithms, namely TGBKA, BKA, SCGJO, COA, SABO, and HHO, on the CEC2017 test set. The x-axis in Figure 3 represents the number of iterations, and the y-axis represents the fitness function. Find the minimum value of the function, and the smaller the value, the better the performance of the algorithm.

Unimodal functions are mainly used to test the local exploitation ability of the algorithm. Except for F2, which was removed by the official due to problems, TGBKA obtained the best results in F1 and F3 in the convergence curves. The growth rate of the optimization accuracy of TGBKA in the early stage is faster than that of other algorithms in terms of magnitude, and its optimization accuracy is also better than that of other algorithms.

Multimodal functions are mainly used to test the global exploration ability and the ability to escape from local optima of the algorithm. TGBKA found the optimal values in F6, F7, F8, F9, and F10, indicating that the algorithm can effectively avoid local optima. Hybrid functions are mainly used to evaluate the robustness in multimodal environments. TGBKA obtained the optimal values in ten functions, including F11, F12, F13, F14, F15, F16, F17, F18, F19, and F20, indicating that the algorithm has strong anti-interference ability and environmental adaptability. Composite functions usually integrate multiple sub-functions through rotation, translation, weighting, etc., forming a complex solution space structure. Their global optimum may be hidden in the “traps” of multiple local optima. The results can reflect the comprehensive performance of the optimization algorithm in solving highly complex, non-linear, and multimodal problems. The results show that the algorithm can obtain the optimal solutions in F21, F22, F24, F25, F27, F28, F29, and F30, indicating that the algorithm has the ability to escape from local optima and explore complex structures.

Through this detailed simulation experiment analysis, the effectiveness of the Tent chaos mapping and Gaussian mutation in escaping local optima and fast search is further verified, and it is shown that the TGBKA algorithm has strong optimization ability and convergence speed. It also provides strong support and reference for solving practical optimization problems.

### 6.4. UAV Path Optimization Comparison

#### 6.4.1. Simulation Scene 1

In this simulation test, the flight space is configured with dimensions of 1000 m × 1000 m × 400 m. The population size is set as *n_Pop_* = 100, and the maximum number of iterations is designated as *MaxIt* = 10. Five algorithms are chosen for comparison, namely the basic Black-winged Kite Algorithm (BKA), the Sine Cosine Golden Jackal Optimization Algorithm (SCGJO), the Coati Optimization Algorithm (COA), the Subtraction Averaging Optimizer (SABO), and the Harris Hawks Optimization Algorithm (HHO). During the flight process, the Unmanned Aerial Vehicle (UAV) is required to navigate from the starting point to the destination while avoiding threat sources. The performance of each algorithm in UAV path planning is evaluated using a cost function; specifically, a lower cost indicates superior performance. This experiment utilizes two distinct scenarios to assess how well each algorithm performs in UAV path planning tasks.

**Figure 4:**
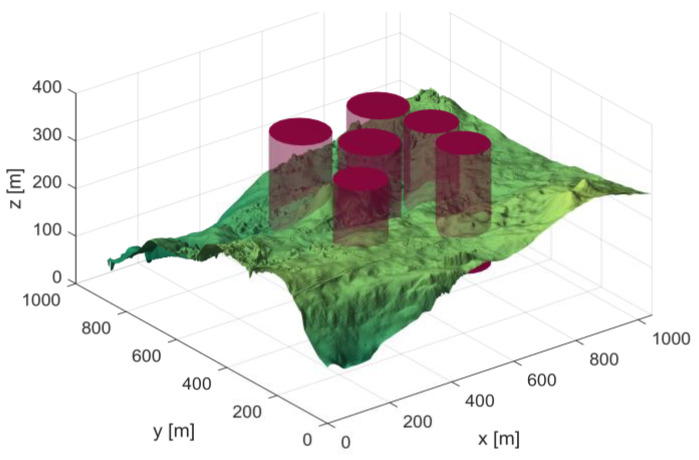
3D Scene Map 1.

In Scenario 1, there are six threat sources, and the details of these threat sources are presented in Table 2. The UAV initiates its flight from the position (200, 100, 150) and aims to reach the end position at (800, 800, 150). Figure 4 depicts the three-dimensional scene of Scenario 1.

**Figure 5:**
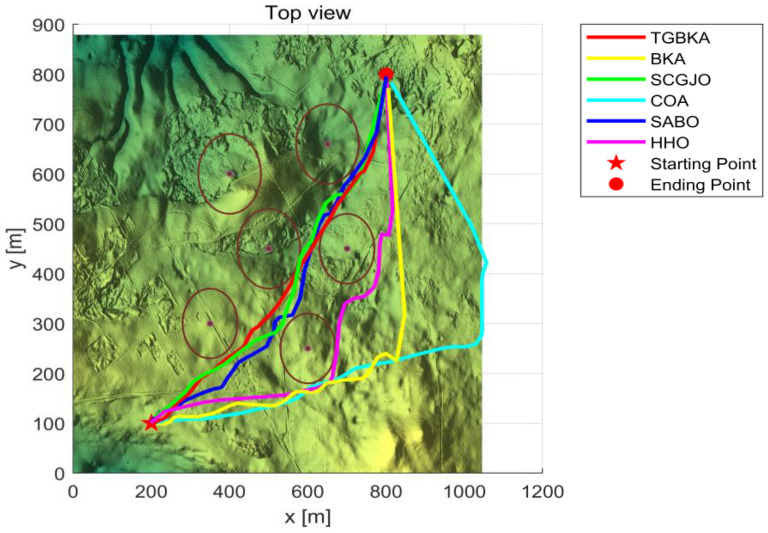
Top-view of UAV Path Planning in Scene 1.

**Figure 6:**
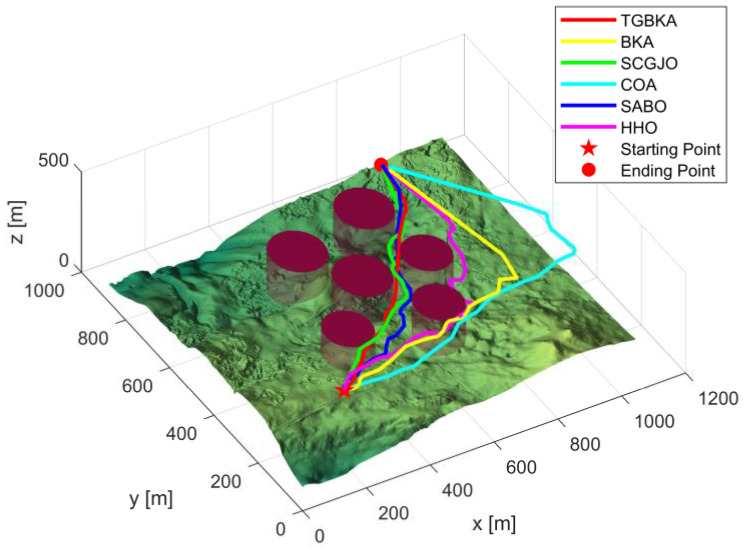
3D Simulation Map of UAV Path Planning in Scene 1.

**Figure 7:**
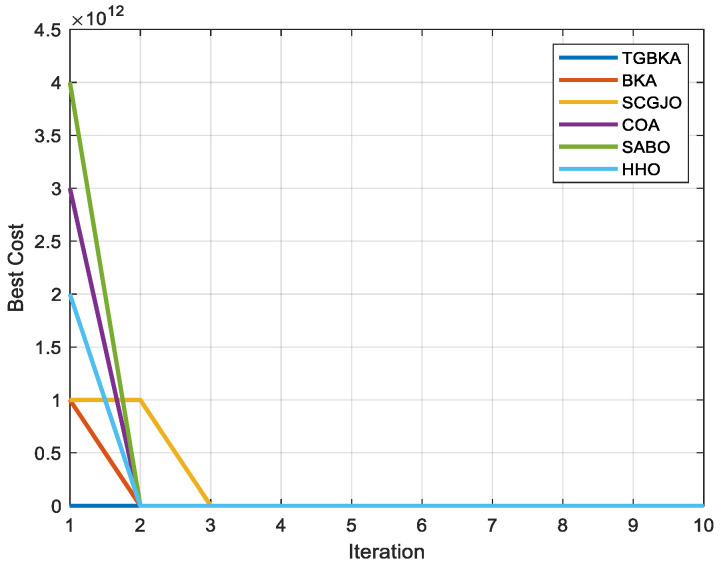
UAV Iteration Curve of Scene 1.

In Scene 1, Figure 5 and Figure 6 display the top-view and 3D simulation of the UAV path planning for each algorithm, respectively. As is evident from these figures, the TGBKA outperforms other algorithms in both the top-view and 3D coordinate representations. The COA, BKA, and HHO algorithms produce redundant trajectories, thereby increasing the length of the planned path. Additionally, the path of the BKA is not smooth, featuring excessive fluctuations in the flight angle. The UAV iteration curve in Figure 7 further reveals that the TGBKA algorithm remarkably enhances the algorithm’s optimization capacity and guarantees its convergence speed. When considered in conjunction with Figure 5 and Figure 6, this evidence validates the TGBKA’s proficiency in path planning within complex environments.

#### 6.4.2. Simulation Scene 2

**Figure 8:**
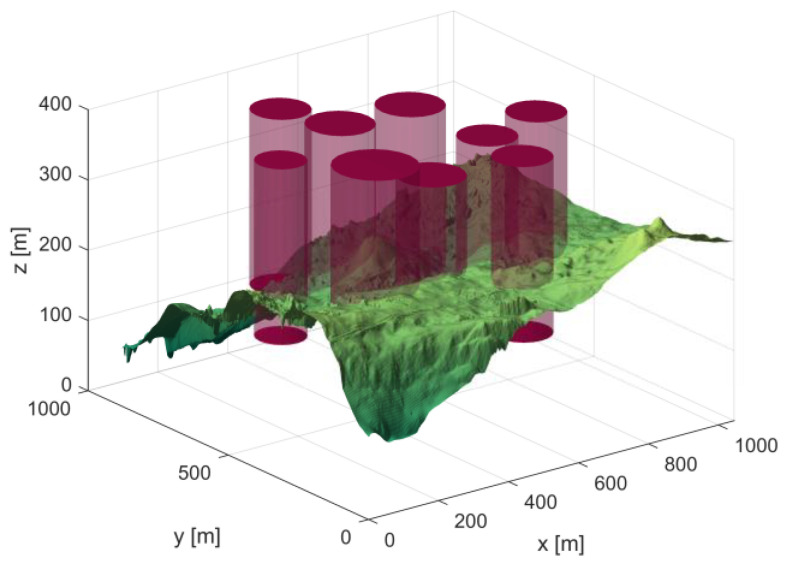
3D Scene Map 2.

In Scene 2, there are nine threat sources, and the threat source information is shown in Table 3. The starting position of the UAV is (200, 100, 150), and the end position is (800, 800, 150). Figure 8 shows the 3D scene after setting the parameters.

**Figure 9:**
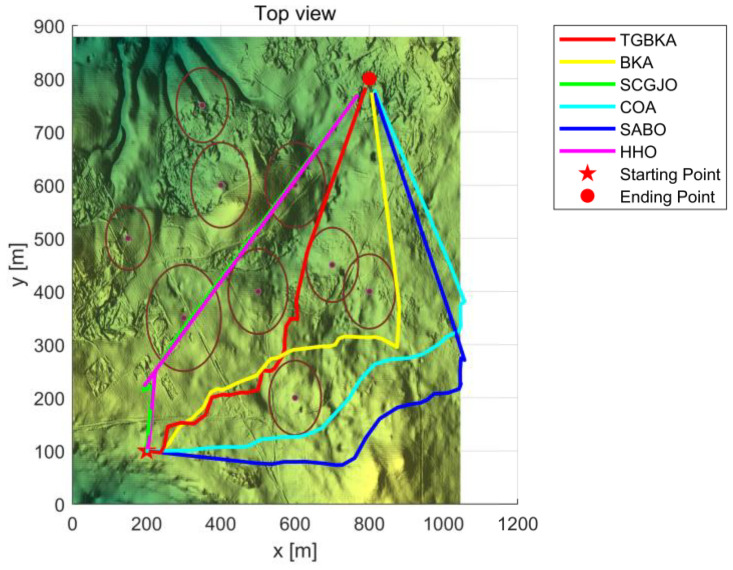
Top-view of UAV Path Planning in Scene 2.

**Figure 10:**
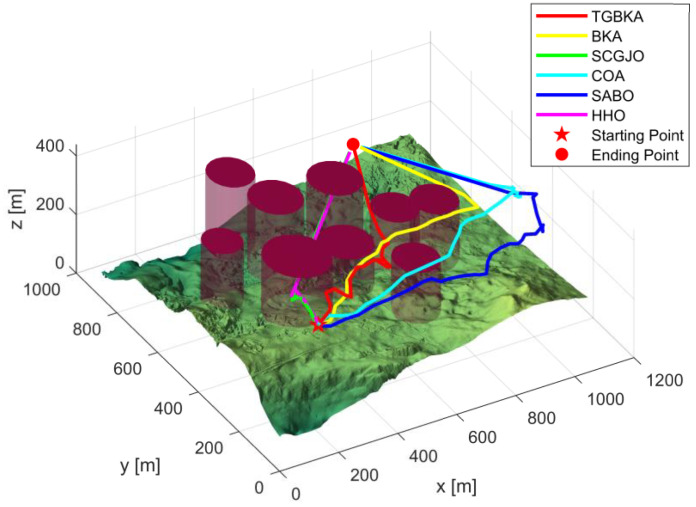
3D Simulation Map of UAV Path Planning in Scene 2.

In Scene 2, the top-view and 3D simulation visualizations of the UAV path planning for each algorithm are meticulously illustrated in Figure 9 and Figure 10, respectively. Upon close examination, it becomes evident that even with the significant increase in the number of threat sources, which inherently makes Scene 2 far more complex and challenging than Scene 1, the TGBKA algorithm remains unwavering in its performance. It consistently demonstrates its remarkable superiority in efficiently identifying and traversing the shortest and most optimal path.

**Figure 11:**
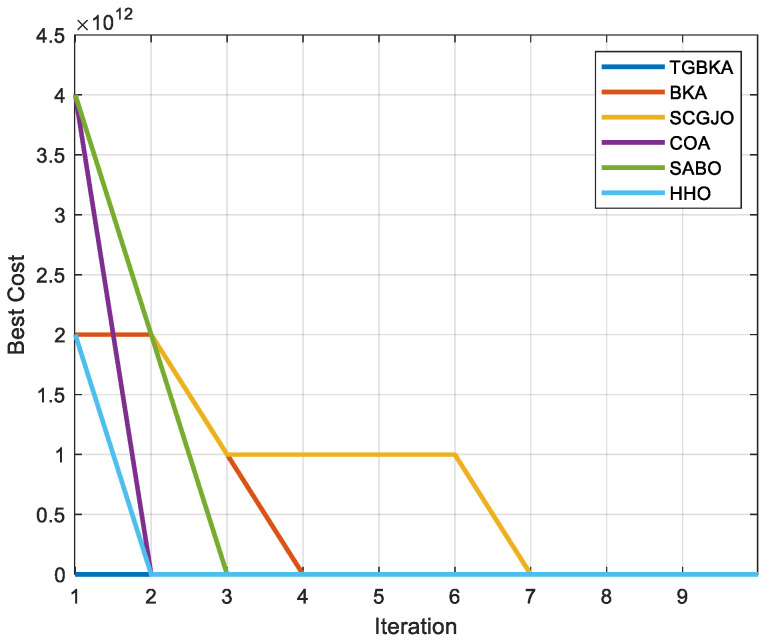
UAV Iteration Curve of Scene 2.

In Figure 9, a striking contrast is observed among the algorithms. The SCGJO and HHO algorithms, unfortunately, fail to adapt to the hazardous environment and are unable to avoid the threat sources. Instead, they take the risky route of flying directly through them, which not only compromises the safety of the UAV but also reflects poorly on their path-planning capabilities. In contrast, the BKA, SABO, and COA algorithms do recognize the threat sources and attempt to navigate around them. However, their detours are suboptimal, leading to a substantial increase in the path length. This unnecessary elongation of the route not only consumes more resources but also significantly affects the overall cost of the operation, highlighting the limitations of these algorithms in handling complex scenarios. Figure 10 presents the iteration cost curve of each algorithm in Scene 2, providing a quantitative analysis of their performance over time. Meanwhile, Figure 11 showcases the UAV iteration curve for the same scene, which vividly depicts the progress and efficiency of each algorithm in finding the optimal path.

It is abundantly clear that the improved TGBKA algorithm has successfully overcome several long-standing issues that have plagued traditional algorithms, such as slow convergence speed, insufficient precision in results, and notably weak optimization capabilities in the later stages of the process. This advanced algorithm has proven itself to be highly versatile, capable of performing accurate and efficient path planning in both simple, straightforward environments and complex, dynamic scenarios filled with numerous obstacles and uncertainties. Its superior performance is further emphasized by its relatively short execution time, making it a highly practical and efficient solution for UAV path planning. The convergence curves mentioned above offer a comprehensive and detailed view of the performance of different algorithms throughout the iteration process. At the very beginning of the iteration, the TGBKA algorithm immediately stands out with its exceptional convergence speed, outpacing all the other algorithms. Following closely behind are HHO, BKA, COA, SABO, and SCGJO. However, as the iterations progress, it becomes apparent that the SCGJO algorithm struggles to achieve high convergence accuracy in the later stages, indicating its limitations in complex optimization tasks. As the number of iterations continues to increase, the convergence accuracy of HHO gradually approaches that of TGBKA. Yet, what truly sets the TGBKA algorithm apart is its consistent and unwavering performance. It maintains its leading position in iteration performance throughout the entire process, clearly demonstrating its robustness and reliability. Therefore, it can be firmly concluded that the TGBKA algorithm performs outstandingly well in terms of both convergence speed and accuracy during the iteration process, making it a top-tier choice for UAV path-planning applications.

In summary, after a thorough and comprehensive comparison with the other five algorithms, it is undeniable that the TGBKA algorithm can achieve outstanding optimization results when dealing with the UAV path-planning problem in complex environments. It possesses a unique ability to better address the notorious local-optimum problem that often traps traditional algorithms, enabling it to explore the solution space more effectively. With its faster convergence speed, it can quickly home in on the optimal solution, saving valuable time and resources. Moreover, in the challenging context of complex environments, it can effectively shorten the flight distance of the UAV, ensuring more efficient and safer operations. This makes the TGBKA algorithm a highly promising and practical solution for a wide range of UAV path-planning tasks.

While in flight, the Unmanned Aerial Vehicle (UAV) is required to navigate from the initial position to the final destination, all the while avoiding threat sources along the way. To assess how well each algorithm performs in UAV path planning, a cost function is employed. The performance of an algorithm is directly related to the value of this cost function; specifically, a lower cost value signifies superior performance. In order to thoroughly evaluate the performance of each algorithm in UAV path planning, this experiment utilizes two distinct scenarios.

## 7. Conclusions

Traditional optimization algorithms frequently encounter difficulties when attempting to solve complex optimization problems. These complex problems, which often involve high-dimensional spaces, non-linear relationships, and multiple objectives, pose significant challenges to the capabilities of traditional algorithms. In contrast, swarm intelligence algorithms have gained considerable popularity. Their appeal lies in their straightforward implementation and relatively low computational requirements, making them more adaptable to a wide range of complex scenarios compared to traditional counterparts. In this paper, the focus is on the research and enhancement of the Black-winged Kite Optimization Algorithm, and the key contributions are outlined as follows: The basic Black-winged Kite Optimization Algorithm faces several inherent issues during its iterative process. Notably, it suffers from a lack of population diversity, which limits its ability to explore the solution space comprehensively. Additionally, it has a pronounced tendency to get trapped in local optima, preventing it from reaching the global optimal solution. To address these shortcomings, this paper innovatively integrates two effective strategies. The Tent chaos mapping is introduced during the initialization of the black-winged kite population. This approach helps to generate an initial population that is more evenly distributed across the solution space, enhancing the algorithm’s exploration capabilities from the start. Meanwhile, Gaussian mutation is incorporated into the migration stage of the black-winged kites. This mutation strategy enables the algorithm to escape from local optima more effectively and improve its convergence speed. As a result, an improved Black-winged Kite Optimization Algorithm (TGBKA) that combines these multiple strategies is proposed. To thoroughly assess the performance of the TGBKA, a series of comparative optimization experiments are conducted. The TGBKA is compared with 5 similar optimization algorithms, including the original BKA and SCGJO, using 29 functions from the CEC2017 benchmark function test set. This test set is widely recognized for its comprehensiveness in representing various types of optimization problems. The experimental results are highly revealing. Compared to other algorithms, the TGBKA demonstrates remarkable superiority in multiple aspects. It exhibits a significantly faster optimization speed in the early stages, allowing it to quickly converge towards promising regions of the solution space. In terms of accuracy, it can obtain more precise solutions. Its convergence speed is also much faster, reducing the overall computational time required to reach the optimal solution. Moreover, the TGBKA effectively overcomes the issue of being trapped in local optima. In multimodal environments, which are particularly challenging for optimization algorithms, its robustness outperforms that of the other comparison algorithms. It also shows strong capabilities in escaping from local optima and exploring complex solution structures, making it a highly reliable choice for complex optimization tasks.

The promising results of the TGBKA in UAV path planning have far-reaching implications for future research in this field. One of the key areas that can benefit from these findings is multi-agent UAV coordination. In scenarios where multiple UAVs need to operate in tandem, such as in large-scale surveillance, search and rescue missions, or package delivery networks, efficient path planning for each individual UAV while ensuring seamless coordination among them is crucial. The TGBKA’s ability to find optimal paths in complex environments can be extended to multi-agent systems. By integrating principles of distributed optimization and communication protocols, multiple UAVs equipped with the TGBKA can dynamically adjust their paths in real-time to avoid collisions, optimize overall mission efficiency, and adapt to changing environmental conditions. For instance, in a search and rescue operation, UAVs could use the TGBKA to independently navigate through challenging terrains filled with obstacles while coordinating with each other to cover the search area as comprehensively and quickly as possible. Another important aspect is dynamic obstacle avoidance. In real-world scenarios, UAVs often encounter unexpected obstacles that emerge during flight, such as sudden changes in weather conditions causing debris to appear or the movement of other airborne objects. The TGBKA’s robustness and ability to escape from local optima make it well-suited for handling such dynamic situations. Future research could focus on integrating real-time sensing data with the TGBKA, enabling UAVs to continuously update their path plans in response to new obstacles. This would require the development of algorithms that can quickly re-evaluate the solution space and find alternative optimal paths without sacrificing too much in terms of efficiency and accuracy.

Furthermore, the TGBKA’s performance in complex environments opens up possibilities for its application in more challenging and specialized UAV missions. For example, in urban environments with numerous buildings, communication towers, and restricted airspace, UAVs need to navigate with high precision. The TGBKA could be further refined to incorporate additional constraints and factors specific to urban settings, such as no-fly zones, air traffic regulations, and the need to avoid disturbing residents. In addition, for long-range UAV missions where energy consumption is a critical factor, the TGBKA could be optimized to find paths that minimize energy usage while still ensuring timely arrival at the destination. Overall, the TGBKA serves as a solid foundation for future research and development in UAV path optimization, offering new directions and opportunities for enhancing the capabilities of UAVs in a wide range of applications.

## Figures and Tables

**Table 1:** Comparison of Optimization Results of Test Functions.

Function	Indicator	TGBKA	BKA	SCGJO	COA	SABO	HHO
F1	Min	1.8 × 10^9^	2.84 × 10^10^	1.16 × 10^10^	3.77 × 10^10^	5.83 × 10^9^	6.92 × 10^7^
	Mean	6.57 ×10^9^	4.54 × 10^10^	2.58 × 10^10^	5.69 × 10^10^	1.48 × 10^10^	4.74 × 10^8^
	Std	3.11 × 10^9^	8.58 × 10^9^	7.58 × 10^9^	8.44 × 10^9^	6.40 × 10^9^	4.60 × 10^8^
F2	Min	0	0	0	0	0	0
	Mean	0	0	0	0	0	0
	Std	0	0	0	0	0	0
F3	Min	1.85 × 10^4^	6.76 × 10^4^	5.95 × 10^4^	7.27 × 10^4^	4.76 × 10^4^	3.96 × 10^4^
	Mean	2.99 × 10^4^	1.05 × 10^5^	7.69 × 10^4^	8.38 × 10^4^	6.58 × 10^4^	5.59 × 10^4^
	Std	7.91 × 10^3^	2.58 × 10^4^	7.33 × 10^3^	4.63 × 10^3^	9.24 × 10^3^	6.96 × 10^3^
F4	Min	5.95 × 10^2^	4.68 × 10^3^	9.04 × 10^2^	1.25 × 10^4^	9.07 × 10^2^	5.98 × 10^2^
	Mean	1.08 × 10^3^	9.40 × 10^3^	4.00 × 10^3^	1.59 × 10^4^	2.02 × 10^3^	7.35 × 10^2^
	Std	4.51 × 10^2^	2.95 × 10^3^	2.62 × 10^3^	1.79 × 10^3^	6.93 × 10^2^	8.79 × 10^1^
F5	Min	6.38 × 10^2^	7.15 × 10^2^	7.40 × 10^2^	8.20 × 10^2^	7.45 × 10^2^	6.86 × 10^2^
	Mean	7.06 × 10^2^	7.79 × 10^2^	8.26 × 10^2^	9.22 × 10^2^	8.07 × 10^2^	7.60 × 10^2^
	Std	2.79 × 10^1^	3.61 × 10^1^	4.44 × 10^1^	3.17 × 10^1^	3.35 × 10^1^	3.33 × 10^1^
F6	Min	6.33 × 10^2^	6.53 × 10^2^	6.54 × 10^2^	6.74 × 10^2^	6.48 × 10^2^	6.57 × 10^2^
	Mean	6.56 × 10^2^	6.69 × 10^2^	6.74 × 10^2^	6.91 × 10^2^	6.68 × 10^2^	6.68 × 10^2^
	Std	6.90	6.46	9.46	6.85	1.13×10^1^	4.91
F7	Min	1.05 × 10^3^	1.09 × 10^3^	1.13 × 10^3^	1.24 × 10^3^	1.06 × 10^3^	1.11 × 10^3^
	Mean	1.16 × 10^3^	1.24 × 10^3^	1.27 × 10^3^	1.42 × 10^3^	1.13 × 10^3^	1.32 × 10^3^
	Std	5.63 × 10^1^	1.18 × 10^2^	7.15 × 10^1^	5.85 × 10^1^	4.95 × 10^1^	8.09 × 10^1^
F8	Min	9.27 × 10^2^	9.55 × 10^2^	1.01 × 10^3^	1.11 × 10^3^	1.02 × 10^3^	9.50 × 10^2^
	Mean	9.60 × 10^2^	1.03 × 10^3^	1.07 × 10^3^	1.14 × 10^3^	1.09 × 10^3^	9.87 × 10^2^
	Std	1.96 × 10^1^	3.30 × 10^1^	3.89 × 10^1^	1.75 × 10^1^	3.06 × 10^1^	2.25 × 10^1^
F9	Min	2.39 × 10^3^	4.11 × 10^3^	5.99 × 10^3^	8.57 × 10^3^	3.26 × 10^3^	7.05 × 10^3^
	Mean	4.73 × 10^3^	6.30 × 10^3^	9.63 × 10^3^	1.10 × 10^4^	7.65 × 10^3^	8.47 × 10^3^
	Std	8.01 × 10^2^	1.07 × 10^3^	1.84 × 10^3^	1.37 × 10^3^	2.31 × 10^3^	8.99 × 10^2^
F10	Min	4.14 × 10^3^	5.49 × 10^3^	6.23 × 10^3^	8.23 × 10^3^	8.11 × 10^3^	4.66 × 10^3^
	Mean	5.18 × 10^3^	5.90 × 10^3^	7.58 × 10^3^	8.97 × 10^3^	8.81 × 10^3^	6.12 × 10^3^
	Std	4.56 × 10^2^	2.39 × 10^2^	7.12 × 10^2^	3.56 × 10^2^	3.98 × 10^2^	7.93 × 10^2^
F11	Min	1.31 × 10^3^	3.34 × 10^3^	2.51 × 10^3^	4.43 × 10^3^	2.66 × 10^3^	1.35 × 10^3^
	Mean	1.47 × 10^3^	1.19 × 10^4^	7.01 × 10^3^	9.26 × 10^3^	5.61 × 10^3^	1.56 × 10^3^
	Std	1.47 × 10^2^	5.92 × 10^3^	2.39 × 10^3^	2.42 × 10^3^	1.73 × 10^3^	1.69 × 10^2^
F12	Min	1.58 × 10^6^	2.76 × 10^9^	4.20 × 10^8^	5.85 × 10^9^	1.21 × 10^8^	1.07 × 10^7^
	Mean	8.73 × 10^7^	7.53 × 10^9^	3.52 × 10^9^	1.31 × 10^10^	6.96 × 10^8^	7.94 × 10^7^
	Std	1.40 × 10^8^	2.02 × 10^9^	2.16 × 10^9^	3.13 × 10^9^	6.68 × 10^8^	4.48 × 10^7^
F13	Min	5.13 × 10^4^	7.08 × 10^7^	2.06 × 10^7^	2.51 × 10^9^	3.44 × 10^6^	3.56 × 10^5^
	M×10an	1.65 × 10^5^	2.88 × 10^9^	1.55 × 10^9^	8.79 × 10^9^	2.00 × 10^8^	1.03 × 10^6^
	Std	9.02 × 10^4^	2.14 × 10^9^	1.96 × 10^9^	4.41 × 10^9^	4.32 × 10^8^	9.20 × 10^5^
F14	Min	1.62 × 10^3^	2.13 × 10^4^	5.50 × 10^4^	9.27 × 10^4^	4.65 × 10^4^	5.03 × 10^4^
	Mean	5.88 × 10^3^	2.28 × 10^6^	1.98 × 10^6^	4.74 × 10^6^	1.14 × 10^6^	1.22 × 10^6^
	Std	6.14 × 10^3^	2.49 × 10^6^	1.10 × 10^6^	4.14 × 10^6^	1.10 × 10^6^	1.25 × 10^6^
F15	Min	1.30 × 10^4^	1.23 × 10^4^	1.98 × 10^5^	2.88 × 10^7^	7.44 × 10^4^	3.28 × 10^4^
	Mean	2.89 × 10^4^	5.16 × 10^7^	1.68 × 10^8^	8.85 × 10^8^	3.28 × 10^6^	1.02 × 10^5^
	Std	2.21 × 10^4^	7.87 × 10^7^	2.12 × 10^8^	4.99 × 10^8^	5.06 × 10^6^	6.22 × 10^4^
F16	Min	2.37 × 10^3^	3.97 × 10^3^	2.63 × 10^3^	4.26 × 10^3^	3.46 × 10^3^	2.90 × 10^3^
	Mean	3.05 × 10^3^	5.24 × 10^3^	3.85 × 10^3^	5.95 × 10^3^	4.21 × 10^3^	3.64 × 10^3^
	Std	3.68 × 10^2^	1.10 × 10^3^	5.10 × 10^2^	9.96 × 10^2^	3.40 × 10^2^	3.96 × 10^2^
F17	Min	1.83 × 10^3^	2.32 × 10^3^	2.24 × 10^3^	2.61 × 10^3^	2.69 × 10^3^	2.18 × 10^3^
	Mean	2.22 × 10^3^	3.24 × 10^3^	2.75 × 10^3^	5.24 × 10^3^	2.98 × 10^3^	2.63 × 10^3^
	Std	2.03 × 10^2^	1.94 × 10^3^	3.87 × 10^2^	3.10 × 10^3^	2.90 × 10^2^	2.48 × 10^2^
F18	Min	2.97 × 10^4^	1.95 × 10^5^	7.29 × 10^5^	1.18 × 10^6^	3.67 × 10^5^	4.42 × 10^5^
	Mean	1.29 × 10^5^	3.82 × 10^7^	1.01 × 10^7^	6.52 × 10^7^	4.67 × 10^6^	3.99 × 10^6^
	Std	1.16 × 10^5^	6.46 × 10^7^	7.92 × 10^6^	4.46 × 10^7^	5.49 × 10^6^	3.77 × 10^6^
F19	Min	1.66 × 10^4^	8.47 × 10^4^	1.29 × 10^6^	6.04 × 10^7^	4.00 × 10^5^	7.42 × 10^4^
	Mean	2.16 × 10^5^	7.84 × 10^7^	3.79 × 10^8^	7.33 × 10^8^	7.08 × 10^6^	1.34 × 10^6^
	Std	3.06 × 10^5^	1.45 × 10^8^	5.36 × 10^8^	3.83 × 10^8^	6.79 × 10^6^	1.17 × 10^6^
F20	Min	2.30 × 10^3^	2.47 × 10^3^	2.63 × 10^3^	2.63 × 10^3^	2.73 × 10^3^	2.38 × 10^3^
	Mean	2.46 × 10^3^	2.78 × 10^3^	3.00 × 10^3^	3.04 × 10^3^	3.11 × 10^3^	2.78 × 10^3^
	Std	1.14 × 10^2^	1.45 × 10^2^	2.27 × 10^2^	1.74 × 10^2^	1.59 × 10^2^	2.38 × 10^2^
F21	Min	2.25 × 10^3^	2.50 × 10^3^	2.51 × 10^3^	2.65 × 10^3^	2.54 × 10^3^	2.29 × 10^3^
	Mean	2.51 × 10^3^	2.61 × 10^3^	2.58 × 10^3^	2.76 × 10^3^	2.61 × 10^3^	2.58 × 10^3^
	Std	6.22 × 10^1^	5.99 × 10^1^	4.43 × 10^1^	5.14 × 10^1^	3.44 × 10^1^	7.09 × 10^1^
F22	Min	2.55 × 10^3^	6.34 × 10^3^	3.63 × 10^3^	7.28 × 10^3^	3.18 × 10^3^	2.65 × 10^3^
	Mean	3.65 × 10^3^	7.56 × 10^3^	5.59 × 10^3^	9.45 × 10^3^	4.49 × 10^3^	6.94 × 10^3^
	Std	1.26 × 10^3^	4.90 × 10^2^	1.24 × 10^3^	9.10 × 10^2^	7.32 × 10^2^	1.33 × 10^3^
F23	Min	2.88 × 10^3^	3.22 × 10^3^	2.88 × 10^3^	3.34 × 10^3^	3.05 × 10^3^	3.03 × 10^3^
	Mean	3.10 × 10^3^	3.72 × 10^3^	2.96 × 10^3^	3.69 × 10^3^	3.19 × 10^3^	3.24 × 10^3^
	Std	1.22 × 10^2^	1.89 × 10^2^	5.22 × 10^1^	1.76 × 10^2^	9.10 × 10^1^	1.48 × 10^2^
F24	Min	3.07 × 10^3^	3.65 × 10^3^	2.99 × 10^3^	3.47 × 10^3^	3.14 × 10^3^	3.21 × 10^3^
	Mean	3.28 × 10^3^	4.12 × 10^3^	3.10 × 10^3^	3.80 × 10^3^	3.26 × 10^3^	3.47 × 10^3^
	Std	1.22 × 10^2^	2.43 × 10^2^	6.07 × 10^1^	1.12 × 10^2^	8.81 × 10^1^	1.33 × 10^2^
F25	Min	2.98 × 10^3^	3.45 × 10^3^	3.28 × 10^3^	4.17 × 10^3^	3.08 × 10^3^	2.93 × 10^3^
	Mean	3.09 × 10^3^	4.07 × 10^3^	3.82 × 10^3^	5.20 × 10^3^	3.41 × 10^3^	3.02 × 10^3^
	Std	8.56 × 10^1^	2.54 × 10^2^	2.86 × 10^2^	4.75 × 10^2^	1.38 × 10^2^	3.85 × 10^1^
F26	Min	4.08 × 10^3^	7.76 × 10^3^	4.38 × 10^3^	9.95 × 10^3^	7.05 × 10^3^	7.14 × 10^3^
	Mean	6.76 × 10^3^	9.95 × 10^3^	7.81 × 10^3^	1.16 × 10^4^	8.42 × 10^3^	8.14 × 10^3^
	Std	1.41 × 10^3^	1.13 × 10^3^	1.33 × 10^3^	8.63 × 10^2^	6.29 × 10^2^	5.69 × 10^2^
F27	Min	3.25 × 10^3^	3.72 × 10^3^	3.27 × 10^3^	3.56 × 10^3^	3.28 × 10^3^	3.26 × 10^3^
	Mean	3.41 × 10^3^	4.56 × 10^3^	3.36 × 10^3^	4.56 × 10^3^	3.51 × 10^3^	3.55 × 10^3^
	Std	1.11 × 10^2^	3.17 × 10^2^	7.08 × 10^1^	4.63 × 10^2^	1.30 × 10^2^	1.70 × 10^2^
F28	Min	3.40 × 10^3^	4.76 × 10^3^	3.85 × 10^3^	6.42 × 10^3^	3.89 × 10^3^	3.36 × 10^3^
	Mean	3.59 × 10^3^	5.87 × 10^3^	4.78 × 10^3^	7.58 × 10^3^	4.45 × 10^3^	3.48 × 10^3^
	Std	2.15 × 10^2^	5.54 × 10^2^	5.55 × 10^2^	6.42 × 10^2^	3.76 × 10^2^	8.01 × 10^1^
F29	Min	4.04 × 10^3^	4.95 × 10^3^	4.25 × 10^3^	5.98 × 10^3^	4.73 × 10^3^	4.36 × 10^3^
	Mean	4.54 × 10^3^	6.90 × 10^3^	4.87 × 10^3^	8.28 × 10^3^	5.70 × 10^3^	4.95 × 10^3^
	Std	3.10 × 10^2^	1.87 × 10^3^	5.35 × 10^2^	1.97 × 10^3^	6.35 × 10^2^	3.85 × 10^2^
F30	Min	2.09 × 10^5^	1.28 × 10^7^	4.53 × 10^6^	3.50 × 10^8^	8.83 × 10^6^	2.00 × 10^6^
	Mean	2.51 × 10^6^	8.44 × 10^8^	1.42 × 10^8^	1.77 × 10^9^	4.36 × 10^7^	1.00 × 10^7^
	Std	1.86 × 10^6^	7.05 × 10^8^	2.42 × 10^8^	9.10 × 10^8^	2.77 × 10^7^	7.01 × 10^6^

**Table 2:** Threat Source Parameters of the First Scene.

Number	Coordinates	Height	Radius
1	(400, 600, 0)	100	80
2	(600, 250, 0)	150	70
3	(500, 450, 0)	100	80
4	(350, 300, 0)	100	70
5	(700, 450, 0)	100	70
6	(650, 660, 0)	100	80

**Table 3:** Threat source parameters of the second scene.

Number	Coordinates	Height	Radius
1	(400, 600, 0)	150	80
2	(600, 200, 0)	150	70
3	(500, 400, 0)	100	80
4	(300, 350, 0)	150	100
5	(700, 450, 0)	120	70
6	(150, 500, 0)	150	60
7	(350, 750, 0)	150	70
8	(800, 400, 0)	150	70
9	(600, 600, 0)	150	80

## Data Availability

The data that support the findings of this study are available from the corresponding author upon request. There are no restrictions on data availability.
